# Correlates and Consequences of Opioid Misuse among High-Risk Young Adults

**DOI:** 10.1155/2014/156954

**Published:** 2014-11-24

**Authors:** Sheree M. Schrager, Aleksandar Kecojevic, Karol Silva, Jennifer Jackson Bloom, Ellen Iverson, Stephen E. Lankenau

**Affiliations:** ^1^Division of Hospital Medicine, Children's Hospital Los Angeles, 4650 Sunset Boulevard, MS No. 94, Los Angeles, CA 90027, USA; ^2^Division of Health Promotion and Behavioral Science, Graduate School of Public Health, San Diego State University, 9245 Sky Park Court, Suite 100, San Diego, CA 92123, USA; ^3^Department of Psychology, Temple University, 1801 N Broad Street, Philadelphia, PA 19122, USA; ^4^Division of Adolescent Medicine, Children's Hospital Los Angeles, 5000 Sunset Boulevard, Los Angeles, CA 90027, USA; ^5^Department of Pediatrics, Keck School of Medicine, University of Southern California, 1975 Zonal Avenue, Los Angeles, CA 90089, USA; ^6^Department of Community Health and Prevention, School of Public Health, Drexel University, 1505 Race Street, Philadelphia, PA 19102, USA

## Abstract

*Background*. Prescription opioids are the most frequently misused class of prescription drug among young adults aged 18–25, yet trajectories of opioid misuse and escalation are understudied. We sought to model opioid misuse patterns and relationships between opioid misuse, sociodemographic factors, and other substance uses. *Methods*. Participants were 575 young adults age 16–25 who had misused opioids in the last 90 days. Latent class analysis was performed with models based on years of misuse, recency of misuse, and alternate modes of administration within the past 12 months, 3 months, and 30 days. *Results*. Four latent classes emerged that were differentially associated with heroin, cocaine, and methamphetamine use, tranquilizer misuse, daily opioid misuse, and opioid withdrawal. Alternate modes of administering opioids were associated with increased risk for these outcomes. Sociodemographic factors, homelessness, prescription history, and history of parental drug use were significantly associated with riskier opioid misuse trajectories. *Conclusion*. Young adults who reported more debilitating experiences as children and adolescents misused opioids longer and engaged in higher risk alternate modes of administering opioids. Data on decisions both to use and to alter a drug's form can be combined to describe patterns of misuse over time and predict important risk behaviors.

## 1. Introduction

Over the past decade, prescription drug misuse has increased significantly in the U.S. [1, 2] and is most prevalent among young adults 18 to 25 years of age [2, 3]. Prescription opioids, such as hydrocodone and oxycodone, are the most frequently misused class of prescription drug among young adults [2]. Prescription opioids are a particularly important public health concern since opioid misuse is associated with a range of negative health outcomes, including injection drug use [4], drug dependence [2, 5], and fatal overdose [6, 7].

Prescription opioid trajectories among young adults begin with initiation into misuse [8] and include various patterns of misuse over a period of years [9, 10]. Features of opioid use trajectories, including duration of misuse and mode of administration, have been linked to negative outcomes among young adults. Individuals who initiate opioid misuse earlier in their lives or have misused opioids for several years have a greater likelihood of developing a substance abuse disorder [11, 12]. Misusing opioids for a period of years has been linked to transitioning to heroin among young injection drug users (IDUs) [8]. Among adults, a longer duration of opioid misuse has also been associated with increased likelihood of engaging in alternate modes of administering opioids, such as injecting or snorting [13].

Mode of administration is a key feature of opioid misuse. Oral administration (typically swallowing pills whole) is the most common mode of administering opioids among young adults [12]; alternate modes of administration include chewing, smoking, snorting, and injecting [4, 14, 15]. These alternate modes involve users crushing or mashing pills [9, 16], which may increase an opioid's potency [14]. Prescription opioid misusers transition to alternate modes of administration for a range of reasons, including an increased tolerance to opioids [8], increasing frequency of opioid misuse [17], and availability of specific opioid formulations [14, 15]. Alternate modes of administering opioids present increased risks for drug dependence [15], drug overdose [4], and infectious disease [16]. Young adults more frequently report alternate modes of administering opioids compared to older users [13, 15, 18].

Despite the importance of administration mode, only a few studies [4, 9, 19] have examined alternate modes of administering opioids among samples of young adults. The objective of the present study is to describe trajectories of opioid misuse among young adults based upon duration of misuse, recency of misuse, and frequency of alternate modes of administration over the past year. We also examine sociodemographic risk factors and other substance use behaviors associated with these trajectories.

## 2. Materials and Methods

### 2.1. Parent Study

Data for this analysis are part of a larger mixed-methods investigation into prescription drug misuse among especially high-risk users (National Institute on Drug Abuse 1R01DA021299). As much of the available research on prescription drug misuse has been limited to students or older populations and excluded hard to reach high-risk groups, and little is known about the usage patterns, risk factors, or concomitant health concerns of recent prescription drug misusers, this study was funded to examine patterns of prescription drug misuse among high-risk young adults in two large US cities representing distinct local markets for prescription and illicit drugs (New York and Los Angeles). The study focused on youth who may be homeless, polydrug users, and/or injection drug users, as they are known to be at especially high risk for negative health outcomes including drug dependence, drug overdose, violence, victimization, and exposure to bloodborne pathogens. The present analysis reflects the second and third aims of the parent study, describing behavioral practices around prescription opioid administration and identifying the relationship between prescription opioid misuse and use of other controlled substances, including illicit drugs.

### 2.2. Participants

Prescription drug misusers were interviewed in Los Angeles and New York between October 2009 and March 2011. Eligible participants were between 16 and 25 years old and had engaged in misuse of a prescription drug, that is, opioid, tranquilizer, or stimulant, or any combination, at least three times in the last 90 days. “Misuse” was defined as taking a prescription drug “when they were not prescribed for you or that you took only for the experience or feeling it caused” [2, p. 13]. The study was not intended to provide prevalence estimates or describe the general population of prescription drug users; rather, a detailed survey of prescription drug misusers was undertaken to obtain nuanced data describing these particularly high-risk individuals.

Sampling was stratified within each site to enroll three groups of young adults with different risk profiles and access to prescription drugs: participants who reported polydrug use within the past 90 days but neither homelessness nor injection drug use (*n* = 202), participants who reported being homeless in the past 90 days but not injection drug use (*n* = 192), and participants who reported injection drug use in the past 90 days (*n* = 202). Interviewers employed both targeted [20] and chain-referral sampling [21], in combination with recruitment data from earlier project phases [4], to recruit young adults in natural settings such as parks, streets, neighborhoods, and organizations serving at-risk youth (e.g., homeless youth). A brief screening tool was used to determine eligibility, and screened individuals received a $3 gift card. Participants who were qualified and were interviewed received a $25 cash incentive. The electronic survey was administered during face-to-face interviews with eligible participants by one of two interviewers at each site in private offices or natural settings. The study protocol was approved by institutional review boards at Drexel University, Children's Hospital Los Angeles, and National Development and Research Institutes, Inc. (New York).

Across the two sites, 4,432 individuals were screened, 831 (18.8%) met the enrollment criteria, and 618 (74.4%) were interviewed. Twenty-two participants (3.6%) were later excluded after their surveys revealed that they had not misused prescription drugs at least three times in the last 90 days, resulting in 596 completed interviews. Of these, 21 participants had never misused opioids, and thus have neither a misuse trajectory to model nor the concomitant health risks. Therefore, they were excluded from the present analysis, resulting in a final analytic sample of 575 prescription opioid misusers.

### 2.3. Measures

#### 2.3.1. Latent Class Indicators

Latent classes (see Section 3, Statistical Analysis) were estimated using seven indicators comprising duration of opioid misuse career, recency of opioid misuse, and recency of administering opioids via alternate modes. Career duration was calculated as the difference between current age and age at which respondents reported first misusing opioids. Recency of opioid misuse was determined by whether participants had misused opioids within the last 12 months, the last three months, and the last 30 days, coded into dummy variables (1 = yes/0 = no) representing each timeframe. Mode of administration was determined with follow-up questions assessing the method by which respondents had misused opioids. Respondents who reported ingesting opioids orally were considered not to have engaged in an alternate mode administration (chewing was not assessed). Respondents who reported injecting, snorting, and/or smoking an opioid were classified as using an alternate mode of administering opioids [15]. Similar to misuse, recency of alternate mode of administration was assessed within the same three time periods (12 months, three months, and 30 days) and coded into dummy variables within each timeframe (1 = alternate mode/0 = no alternate mode).

#### 2.3.2. Correlates of Latent Classes

Demographic factors included binary indicators of race (1 = white/0 = non-white), gender (1 = male/0 = female or other), gender or sexual identity (1 = lesbian, gay, bisexual, or transgender (LGBT)/0 = heterosexual), socioeconomic status growing up (1 = poor-working class/0 = middle-upper class), current school enrollment (1 = yes/0 = no), and site (1 = New York/0 = Los Angeles). Binary variables indicating higher risk background included whether the participant had ever been in foster care, been homeless, injected any drug, participated in a drug treatment program, or overdosed on any drug. Prescription history was represented by four variables indicating whether the participant had ever been prescribed opioids, tranquilizers, or stimulants and whether someone in their family or household had ever been prescribed opioids. Indicators of parental drug misuse included whether the participant's parent(s) had ever misused prescription drugs, used illicit drugs, sniffed any drug, or injected any drug (1 = yes/0 = no for all variables).

#### 2.3.3. Other Drug Uses

Variables included binary indicators of 30-day drug misuse (misuse of prescription tranquilizers; use of heroin, cocaine, and crystal methamphetamine) and symptoms of opioid dependence (daily opioid use and experience of withdrawal). All drug use variables were coded 1 = yes/0 = no.

## 3. Statistical Analysis

Descriptive statistics were calculated using SPSS version 19. Latent class analysis (LCA) was conducted using Mplus version 6 [22]. LCA is a technique that probabilistically assigns individuals to groups or “classes” on the basis of their scores on a set of indicators. The purpose of the LCA is to describe the constellation of clusters that naturally emerge when the entire sample is classified according to their scores on the set of indicators. In the present analysis, we apply LCA to cross-sectional data describing opioid misuse behavior at three time points in order to approximate different trajectories of opioid use. Each emergent class can then be interpreted according to its profile of expected indicator scores, for example, “consistent high risk” and “decreasing risk.” Latent classification procedures have been used successfully to describe patterns of drug use in other populations [23–25]. After determining the most appropriate latent class structure [26], we conducted auxiliary analysis in Mplus [27] to investigate other recent drug misuse, including prescription tranquilizers, heroin, daily opioid misuse, and withdrawal experiences. Finally, we regressed latent class membership on a series of sociodemographic, prescription-related, family-related, and historical correlates in order to characterize risk factors of opioid misuse trajectories.

## 4. Results

### 4.1. Description of Sample

Descriptive statistics are presented for sociodemographic variables in Table 1 and latent class indicators, correlates, and drug use in Table 2. Because few site differences were previously found among these variables [10], data from both sites were combined to form the analytic sample of 575 opioid misusers. The average age of study participants was 20.9 years (SD = 2.1). A majority of participants were male (66%), non-Hispanic white (56%), and heterosexual (68%); many (73%) had experienced homelessness. Participants had been using opioids for an average of 5.4 years (SD = 3.0), and a majority (74%) had previously been prescribed opioids.

### 4.2. Latent Classes

**Figure 1:**
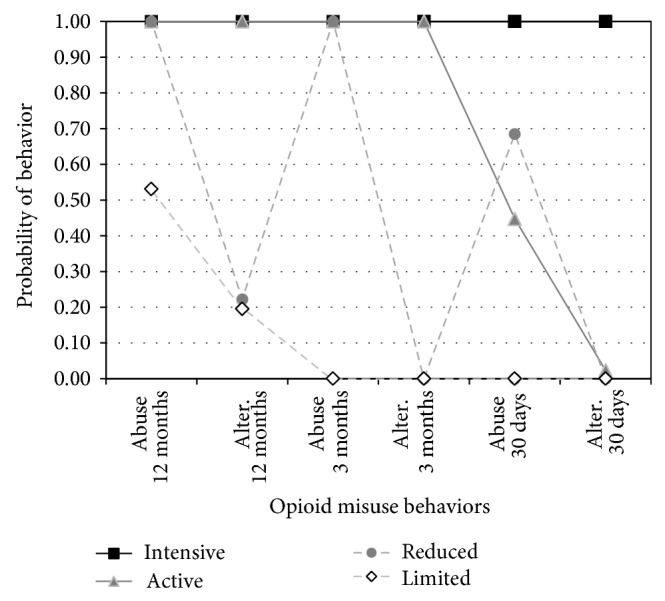
Probabilities of 12-month, 3-month, and 30-day opioid misuse (“abuse”) and alternate modes of administration (“alter”) for each of the four latent classes.

The best-fitting solution resulted in four latent classes (adjusted BIC = 5355.1; entropy = 0.991), the smallest class represented 13.5% of participants. The latent classes, whose opioid misuse and alternate modes of administration are depicted in Figure 1, can be characterized as follows.

#### 4.2.1. Class 1

Class 1 (“intensive users,” 25%) reported the longest duration of opioid misuse of any class (mean = 6.5 years) and consistently misused opioids over the previous 12 months. Intensive users more frequently smoked (27%) or injected (53%) opioids at 12 months than any other class; they also commonly snorted opioids (73%). Intensive users' patterns of smoking, injecting, and snorting remained relatively constant at three months (23%, 49%, and 66%, resp.) but declined somewhat by 30 days (17%, 46%, 59%, resp.). Overall, intensive users' distinctive features include longest duration of opioid misuse, most consistent opioid misuse across all time periods, and consistently engaging in alternate modes (snorting and injecting opioids being the most frequent) across all time periods.

#### 4.2.2. Class 2

Class 2 (“active users,” 13.5%), the smallest class, reported the second longest duration of opioid misuse (mean = 5.7 years). Active users also consistently reported opioid misuse at 12 and 3 months. Compared to intensive users, active users less typically smoked (18%) or injected (25%) opioids but more frequently snorted opioids (84%) at 12 months. Active users' patterns of smoking, injecting, and snorting remained fairly constant at 3 months (13%, 21%, and 80%, resp.) but then reported no alternate modes in the past 30 days, during which time they reported reduced opioid misuse. Overall, active users' distinctive features include consistently engaging in alternate modes (snorting being the most frequent) except for the past 30 days and declining recent opioid misuse.

#### 4.2.3. Class 3

Class 3 (“reduced users,” 44%), the largest group, reported the third longest duration of opioid misuse (mean = 4.9 years). Reduced users reported consistent rates of opioid misuse at 12 and 3 months. However, few smoked, injected, or snorted (3%, 6%, and 16%, resp.) at 12 months, and none reported alternate modes at 3 months or 30 days, indicating that reduced users administered opioids primarily via oral means. Overall, the distinctive features of reduced users are primarily swallowing opioids (minimal use of alternate modes) and declining recent opioid misuse.

#### 4.2.4. Class 4

Class 4 (“limited users,” 17%) reported the shortest duration of opioid misuse (mean = 4.5 years). Limited users had engaged in some opioid misuse in the prior 12 months but had ceased any misuse at least three months prior to the interview. Patterns of smoking, injecting, and snorting, (2%, 6%, and 14%, resp.) at 12 months were similar to the reduced users, which indicates that limited users primarily administered opioids via oral means before cessation at 3 months. Overall, the distinctive features of limited users included: shortest duration of opioid misuse, primarily swallowing opioids (minimal alternate modes), and no misuse in the past 3 months.

### 4.3. Recent Substance Use and Related Risks

The trajectories associated with the latent classes were differentially associated with the likelihood of engaging in recent heroin use (*χ* ^2^(3) = 93.5, *P* < 0.001), cocaine use (*χ* ^2^(3) = 12.9, *P* < 0.01), prescription tranquilizer misuse (*χ* ^2^(3) = 27.2, *P* < 0.001), daily opioid misuse (*χ* ^2^(3) = 64.8, *P* < 0.001), and opioid withdrawal (*χ* ^2^(3) = 52.0, *P* < 0.001). The probabilities of experiencing each outcome corresponding to the four latent classes are presented in Table 3. Intensive users—participants with both consistently high opioid misuse and alternate modes over the past 12 months—had significantly higher probabilities of engaging in recent heroin, cocaine, methamphetamine, and tranquilizer misuse, daily opioid misuse, and experiencing withdrawal from opioids than all other classes. Notably, 30-day heroin use was significantly different between each class, so that the probabilities declined in a stepwise fashion from intensive to limited. Additionally, the probability of daily opioid misuse and opioid withdrawal was significantly higher among the intensive users. There were no significant differences between the other three classes regarding recent misuse of cocaine or tranquilizers.

### 4.4. Correlates of Opioid Misuse Trajectories

In a series of latent class regression analyses, only gender, site, and family prescription history were not associated with any significant differences in likelihood of latent class membership and are not discussed further. The following associations between risk factors and opioid misuse trajectories were found.

#### 4.4.1. Demographics

Participants who are identified as white were less likely to be active users than any other class (intensive: OR = 2.0, *P* < 0.01; reduced: OR = 2.4, *P* < 0.01; limited: OR = 1.9, *P* < 0.05). Those who were identified as LGBT were more likely to be intensive than active users (OR = 1.9, *P* < 0.05), suggesting recent opioid misuse. Participants who reported being poor or working-class growing up were less likely to be limited users than any other class (intensive: OR = 2.6, *P* < 0.001; active: OR = 2.3, *P* < 0.01; reduced: OR = 2.2, *P* < 0.01), whereas current students were more likely to be limited users than any other class (intensive: OR = 0.5, *P* < 0.01; active: OR = 0.3, *P* < 0.001; reduced: OR = 0.1, *P* < 0.001).

#### 4.4.2. Risk Status and Behavior

Participants who reported having been in foster care were more likely to be reduced than limited users (OR = 1.9, *P* < 0.05). Participants who reported ever having been homeless were more likely to be intensive than reduced users (OR = 3.4, *P* < 0.001) and were also more likely to be intensive (OR = 4.4, *P* < 0.001) or active (OR = 2.4, *P* < 0.05) than limited users. Those who had ever injected drugs were more likely to be intensive users than any other class (active: OR = 0.4, *P* < 0.01; reduced: OR = 0.2, *P* < 0.001; limited: OR = 0.2, *P* < 0.001), and they were also more likely to be active than limited users (OR = 2.3, *P* < 0.05). Similarly, participants who had been involved in a drug treatment program were more likely to be intensive users than any other class (active: OR = 0.4, *P* < 0.01; reduced: OR = 0.3, *P* < 0.001; limited: OR = 0.2, *P* < 0.001) and were also more likely to be active users than limited users (OR = 2.0, *P* < 0.05). Finally, participants who had previously overdosed on opioids were more likely to be intensive or active than limited users (intensive: OR = 7.0, *P* < 0.001; active: OR = 4.2, *P* < 0.01) and were also more likely to be intensive than reduced users (OR = 2.9, *P* < 0.001).

#### 4.4.3. Prescription History

Participants who had been prescribed opioids were more likely to be intensive (OR = 1.9, *P* < 0.05) or active (OR = 2.2, *P* < 0.05) than limited users. Those who had been prescribed tranquilizers were more likely to be intensive than reduced (OR = 0.6, *P* < 0.05) or limited users (OR = 0.4, *P* < 0.001). Those who had been prescribed stimulants were more likely to be intensive users than any other class (active: OR = 0.5, *P* < 0.05; reduced: OR = 0.5, *P* < 0.01; limited: OR = 0.6, *P* < 0.01).

#### 4.4.4. Parent Drug Use

Participants whose parents misused prescription drugs of any kind were more likely to be in intensive (OR = 3.0, *P* < 0.001) or reduced users (OR = 2.2, *P* < 0.01) than limited users. Participants were also more likely to be intensive than limited users if their parents had ever misused illicit drugs (OR = 2.2, *P* < 0.01), sniffed drugs (OR = 3.4, *P* < 0.01), or injected drugs (OR = 3.7, *P* < 0.05).

## 5. Discussion

The present study sought to identify common trajectories, or classes, of opioid misuse over the past 12 months in a high-risk sample and determine the correlates of these trajectories and related drug use behavior. Four opioid misuse classes emerged from the LCA analysis. Intensive users reported the longest duration of opioid misuse and the most frequent and diverse alternate modes (primarily snorting and injecting). Active users reported a longer duration of opioid misuse and alternate modes that were more frequent and diverse (primarily snorting). Reduced and limited users reported the shortest duration of opioid misuse and alternate modes of administration that were both infrequent and limited (primarily oral). These findings corroborate research on the adult population of opioid misusers indicating that longer duration of opioid misuse is associated with increased likelihood of engaging in alternate modes of administration [13]. Additionally, our results suggest that longer duration of opioid use is associated with higher-risk alternate modes, such as injection.

Latent classes were also associated with other recent drug use. Intensive users, which consisted of users primarily reporting snorting and injecting opioids, had the highest probability of recent use of heroin, cocaine, methamphetamine, and prescription tranquilizers. These findings corroborate previous qualitative studies showing that tranquilizer misuse is particularly common among young IDUs to boost the effects of opioids and heroin or as a means to self-medicate for opioid withdrawal [4, 28]. Notably, the active, reduced, and limited classes, which evidence a large degree of variability in patterns of opioid misuse but limited injection drug use, showed no differences regarding recent misuse of tranquilizers. The probability of recent heroin use also increased when moving from limited (no recent opioid misuse) to reduced (primarily oral use) to active (primarily snorting) to intensive use (mixture of smoking, injecting, and snorting opioids). Results on heroin use are consistent with previous research [15] reporting increased odds of recent heroin use by opioid injectors and snorters but decreased odds among oral users.

Sociodemographic characteristics, including indicators of race and socioeconomic status, were associated with opioid misuse latent classes. Two important sociodemographic factors, housing status and sexual identity, merit highlighting. Homeless participants were between two and four times as likely to be in the intensive or active use class compared to the limited use class; LGBT participants were nearly twice as likely to be intensive users compared to active users, confirming previous reports from our data on histories of prescription opioid and tranquilizer initiation [29] and raising additional concerns about health outcomes for this group in light of other recent findings associating prescription opioid misuse with unprotected anal intercourse among young men who have sex with men [30]. While both housing status and sexual identity have previously been associated with increased risk for polydrug and illicit drug use [31–33], our results suggest that these statuses are also associated with escalated patterns of opioid misuse. Additionally, participants with parents who misused prescription or illicit drugs, or who snorted or injected drugs, were two to three times as likely to be the high-risk intensive users than other classes. These findings give further support to the potential impact of parental drug use on later drug use [34], including opioid misuse, among young adults. Parental history of prescription drug misuse and a history of being in foster care were also key characteristics distinguishing reduced from limited users, reinforcing the importance of these risk factors and their association with substance use [35] and risk for overdose [36].

Previous studies have reported an association between history of being prescribed drugs and misuse of prescription drugs [37]. Notably, our results indicate that a history of any prescription for opioids, tranquilizers, or stimulants is associated with increased odds of being intensive misusers of opioids. Though the pathway for this relationship (i.e., whether opioid misuse preceded or followed prescription) is unclear, health conditions for which young adults are routinely prescribed opioids, tranquilizers, or stimulants have previously been associated with subsequent patterns of substance misuse [8, 38]. Our findings indicate that individuals with a history of being prescribed medications are more likely to undertake alternate modes of administering opioids, suggesting a potential comorbidity between other health conditions or disorders and more intensive patterns of opioid misuse such as snorting and injecting.

These results provide support for the development and distribution of abuse deterrent formulations (ADFs) of opioids that are resistant to tampering and subsequent smoking, snorting, or injecting [14, 39]. Most of our study data was collected before ADFs, such as OxyContin OP, were introduced in August 2010 and became more widely available in 2011 [40]. Studies indicate that OxyContin has declined as a drug of choice among some opioid users since ADFs were introduced [39]. ADFs may thus help reduce certain patterns of opioid misuse among young adults, including snorting and injecting opioids.

### 5.1. Limitations

The sample is comprised of young adults who were currently homeless and/or had engaged in high-risk behaviors in New York and Los Angeles. By design, this yielded a sample that is not nationally or even locally representative; results may not generalize to populations of young adults who are largely housed, who do not engage in these risk behaviors, or high-risk young adults in other cities or countries. Additionally, although LCA is a sophisticated approach to estimate and describe opioid misuse trajectories over time, the data were cross-sectional, limiting our ability to infer causality, examine the stability of the reported relationships over time, or project participants' future risk behavior based on their current latent class membership. Finally, findings based upon data reflecting events that occurred years prior to being interviewed may be subject to recall or self-report bias. However, most measures concerned events occurring in the past 12 months, and as part of the inclusion criteria all participants demonstrated a willingness to describe their own drug use behavior.

## 6. Conclusions

Young adults in our study who reported more debilitating experiences as children and adolescents misused opioids longer and engaged in higher-risk alternate modes of administering opioids. Recent patterns of substance misuse were most pronounced among these same young adults. Conversely, young adults who reported less risk exposure in their younger years reported less chronic patterns of opioid misuse and less serious patterns of current substance misuse. Our findings highlight the significance of upstream factors on prescription opioid misuse trajectories among high-risk young adults and consequences associated with these trajectories, including drug use practices that increase risk for drug dependence, drug overdose, and infectious disease.

## Figures and Tables

**Table 1:** Demographics of a sample of high-risk prescription opioid misusers (*N* = 575).

Variable	Categories	Mean (SD) or *N* (%)
Age (mean +/− SD)	Range: 16–25	20.89 (2.05)

Race	Non-Hispanic white	320 (55.9%)
	Nonwhite	
	Hispanic	85 (14.8%)
	Multiracial	88 (15.4%)
	Black/African American	61 (10.7%)
	Asian/Pacific Islander	9 (1.6%)
	Native American	7 (1.2%)
	Other	2 (2.3%)

Gender	Male	377 (65.7%)
	Female	182 (31.7%)
	Gender variant	15 (2.6%)

Sexual identity	Heterosexual	388 (67.8%)
	LGBT or questioning	184 (32.2%)
	Gay/lesbian/homosexual	51 (8.9%)
	Bisexual	99 (17.2%)
	Questioning or other	37 (6.4%)

Family poverty growing up	Poor/low Income	258 (45.2%)
	Middle/upper class	313 (54.8%)

Student status	Current student	185 (32.2%)

**Table 2:** Opioid misuse trajectory indicators, risk factors for opioid misuse, and recent drug use and opioid dependence measures (*N* = 575).

	Variable	Mean (SD) or *N* (%)
Latent class/trajectory indicators	Opioid use duration (years)	5.35 (3.001)
	Abused opioids (12 mo)	526 (91.5%)
	Altered form (12 mo)	296 (51.5%)
	Abused opioids (3 mo)	474 (82.4%)
	Altered form (3 mo)	220 (38.3%)
	Abused opioids (30 days)	351 (61.0%)
	Altered form (30 days)	144 (25.0%)

Risk status and behavior	Ever homeless	420 (73.0%)
	Ever in foster care	133 (23.1%)
	Ever injected drugs	249 (43.3%)
	Ever in drug treatment	243 (42.3%)
	Ever overdose on opioids	87 (15.1%)

Prescription history	Ever prescribed opioids	425 (73.9%)
	Family/household ever prescribed opioids	386 (67.1%)
	Ever prescribed tranquilizers	262 (45.6%)
	Ever prescribed stimulants	261 (45.4%)

Parent drug use	Parents misused Rx drugs	189 (32.9%)
	Parents misused illegal drugs	246 (42.8%)
	Parents sniffed drugs	83 (14.4%)
	Parents injected drugs	47 (8.2%)

Other recent drug use	Rx tranquilizer misuse (30 days)	308 (53.6%)
	Heroin use (30 days)	182 (31.7%)
	Cocaine use (30 days)	193 (33.6%)
	Meth use (30 days)	115 (20.0%)

Opioid dependence	Daily opioid misuse (30 days)	288 (50.1%)
	Opioid withdrawal (30 days)	214 (37.2%)

**Table 3:** Probabilities of engaging in or experiencing recent (30-day) drug use outcomes based on prescription opioid misuse trajectories.

Outcome	Class 1	Class 2	Class 3	Class 4
Rx tranquilizer use	0.711^a^	0.508^b^	0.492^b^	0.418^b^
Heroin use	0.607^a^	0.365^b^	0.221^c^	0.111^d^
Cocaine use	0.467^a^	0.300^b^	0.296^b^	0.287^b^
Methamphetamine use	0.275^a^	0.209^a,b^	0.182^b^	0.133^b^
Daily opioid misuse	0.259^a^	0.002^b^	0.066^c^	0.000^b^
Opioid withdrawal	0.293^a^	0.080^b^	0.059^b^	0.010^c^

Note: within a row, probabilities with different superscripts are significantly different from each other.
